# WFIKKN2 is secreted and elevated in blood plasma of HER2-positive breast cancer patients – implications in cancer surveillance and recurrence monitoring

**DOI:** 10.1186/s40364-025-00853-4

**Published:** 2025-11-05

**Authors:** Amir Sabbaghian, Fei Xie, Xiao Fang Wang, Zhen Yang, Ming Chen Zhang, Ting Gang Chew, Shu Wang, Yoon Pin Lim

**Affiliations:** 1 School of Medicine, Zhejiang University-University of Edinburgh Institute, Zhejiang University, Hangzhou, Zhejiang China; 2 Department of Cancer Biology & Innovation, Guoke Ningbo Life Science and Health Industry Research Institute, Ningbo, Zhejiang China; 3 Department of Breast Disease Center, Peking University People’s Hospital, 11 Xizhimen South Street, Beijing, 100044 China; 4 Intelligent Medicine Institute, Shanghai Medical College, Fudan University, Shanghai, China; 5 Department of Endocrinology, Ningbo No.2 Hospital, Ningbo, Zhejiang China; 6 New Materials Innovation Center, National Hi-Tech Industrial Development Zone, Block B2, Juxian Street, Yinzhou District, Ningbo, Zhejiang China

**Keywords:** WFIKKN2, HER2-positive breast cancer, Biomarker, Detection, Surveillance

## Abstract

Current methods for post-treatment cancer surveillance and recurrence monitoring rely mainly on biophysical imaging methods like CT and MRI. Limitations associated with these approaches include risk of radiation, high cost and sophistication in operation. Minimally invasive blood test is a very attractive alternative but there is no biomarker that is of sufficient sensitivity for this purpose. In this study, we attempted to discover novel breast cancer-associated blood plasma proteins that can fill this gap. We tested the hypothesis that genes that are co-amplified HER2 can be used as a surrogate biomarker for detection of HER2+ breast cancer. Following identification of HER2-coamplified genes via copy number variation analysis, a series of bioinformatic tools were used that eventually led to the identification of WFIKKN2 as a novel cancer-associated blood plasma protein. ELISA analysis of more than 120 plasma samples from non-cancer and cancer patients with HER2+ breast cancer revealed WFIKKN2 to have sensitivity and specificity of up to 89% and 60%, respectively. While not ideal as a diagnostic biomarker due to its moderate specificity, the high sensitivity of WFIKKN2 is suitable for the purpose of post-treatment surveillance and recurrence monitoring. The data warrants WFIKKN2 to be further evaluated through clinical studies to validate its clinical utility.

**Supplementary information:**

The online version contains supplementary material available at 10.1186/s40364-025-00853-4.

## To the editor

HER2-positive breast cancer (HER2+ BC) accounts for 15–20% of all breast cancers [1] and has a higher risk of recurrence than other subtypes [2]. Despite advances in HER2-targeted therapies that improved patient outcomes [3], high recurrence rate remains [4]. The standard diagnosis involving biopsy followed by histopathological assessment is unsuitable for post-surgery surveillance [5], while imaging methods such as CT and MRI are constrained by radiation risk, cost, and technical demands [6]. These limitations underscore the need for non-invasive biomarkers to monitor disease progression and recurrence.

**Fig. 1:**
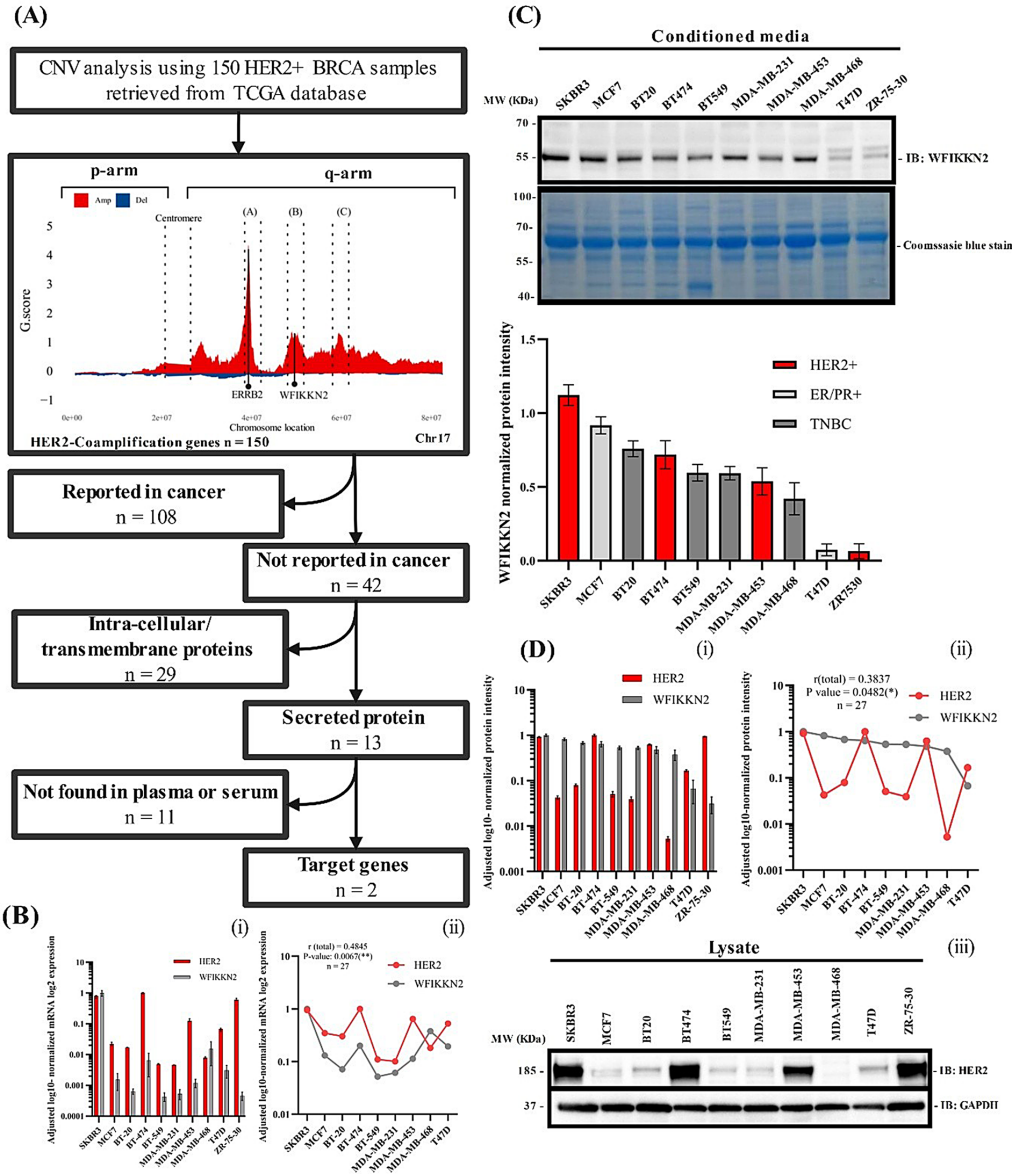
Analysis of HER2-coamplified genes, shortlisting and validation of a selected gene WFIKKN2. **A**, flowchart of CNV meta-analysis for 150 HER2+ BRCA-TCGA samples. A total of 150 genes were identified as co-amplified with HER2 across the q arm of Chr17. Of these, 42 candidate genes have not been previously reported in cancer. Some of these 42 genes are clustered into three distinct regions: region **A** (OSBPL7, LRRC46, PNPO, PRAC2, TTLL6, CALCOCO2, ATP5G1, SNF8, IGF2BP1), region **B** (PHB, AC091180.1, TAC4, EME1, CACNA1G, WFIKKN2, NME1–NME2), and region **C** (SKA2, CTD-2510F5.6, SMG8, YPEL2, PTRH2, TUBD1, RP11-178C3.1, RNFT1, AC025048.1, C17orf64, AC110602.1). Details of the 42 genes and their respective regions are provided in supplementary table S5. Two candidate genes were found following the shortlisting process. The shortlisting process is provided in supplementary information S1. **B**, WFIKKN2 mRNA expression across a panel of BC cell lines (i) and statistical correlation analysis with HER2 mRNA level (ii), total r score = 0.4854 (P-value = 0.007688). **C**, WFIKKN2 protein level in the conditioned media of 10 BC cell lines. Normalization of WFIKKN2 was performed using the strongest Coomassie blue protein band. **D**, HER2 expression in the lysates of a panel of BC cell lines (i) and its correlation analysis with the level of secreted WFIKKN2 (ii). GAPDH was used for HER2 normalization (iii). WFIKKN2 protein expression in cell lysates was not detectable (data not shown). ZR7530 was excluded from the correlation analysis due to its high variability, which was determined to be an outlier based on statistical criteria. n; number of samples, HER2 G.Score ≈4.4, WFIKKN2 G.Score ≈1.3

HER2 amplification occurs within chromosome 17q (C17q), which also harbors other amplified genes [7] that may serve as alternative biomarkers for HER2+ BC. Through copy number variation (CNV) analysis, WFIKKN2 was identified as a novel cancer-associated, secreted plasma protein with potential utility as a surrogate biomarker for HER2 (Fig. 1A). Methods and materials are described in Supplementary Information S1.

WFIKKN2 mRNA level correlated positively with HER2 mRNA in a panel of BC cell lines with a correlation coefficient of about 0.5 (Fig. 1B). Immunoblotting confirmed that secreted WFIKKN2 in the conditioned media correlated with HER2 protein level in cell lysates with modest correlation of about 0.4 (Figs. 1C, 1D). The results show that HER2+ BC cell lines secrete a substantial amount of WFIKKN2, whereas ER/PR+ and triple-negative cell lines also expressed WFIKKN2 at lower levels, suggesting WFIKKN2’s possible role in other breast cancer subtypes that is independent of HER2. A separate study has to be performed to clarify this.

**Fig. 2:**
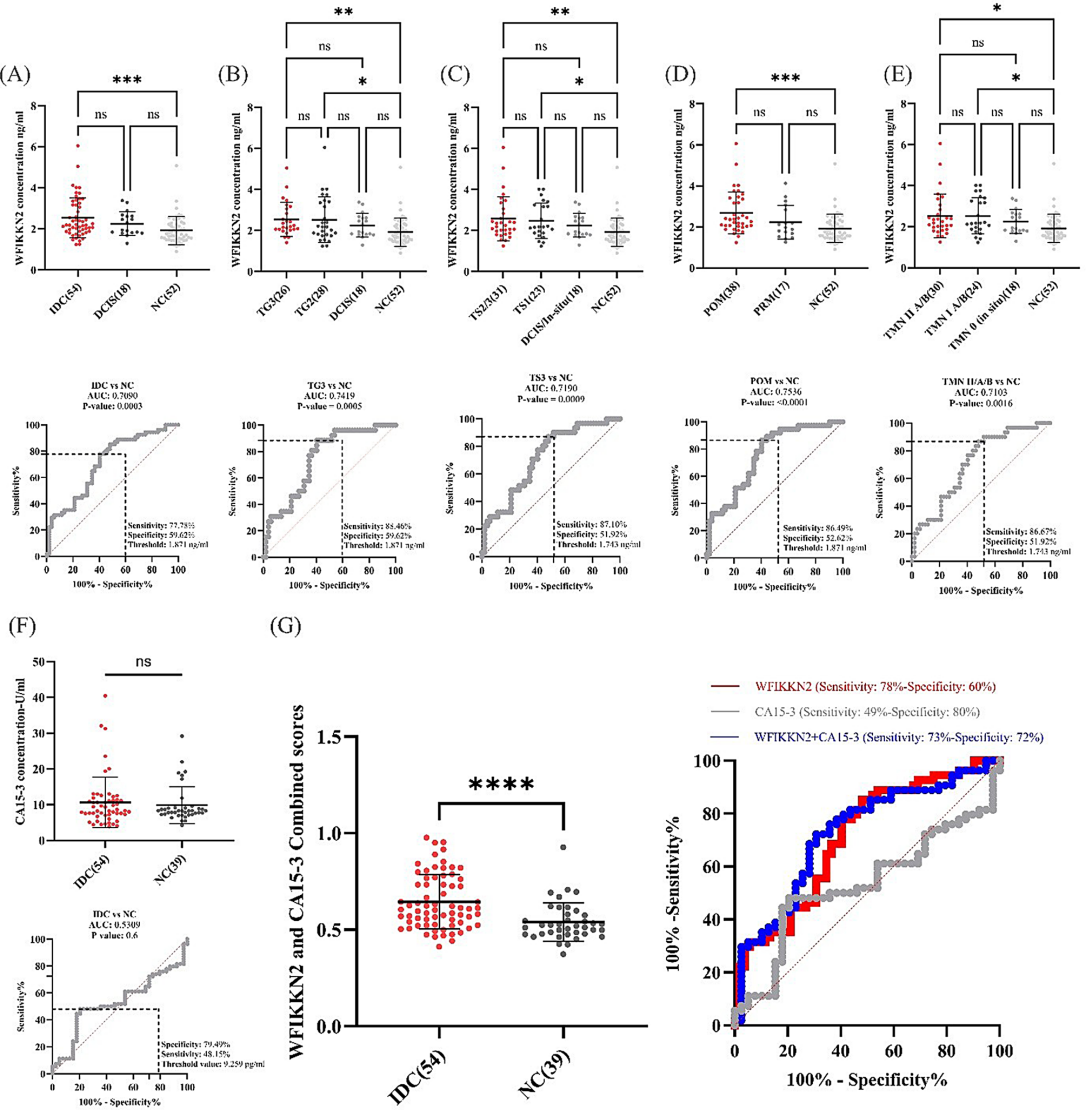
**A–E**, correlation analysis of plasma WFIKKN2 levels (ng/mL) with various histopathological parameters in 72 HER2-positive breast cancer patients comparing to 52 non-cancer controls. **A**, WFIKKN2 abundance in patients with invasive ductal carcinoma (IDC), ductal carcinoma in situ (DCIS), and non-cancer controls (P = 0.0003, AUC = 0.7090, threshold: 1.871 ng/ml). **B**, WFIKKN2 concentration among HER2+ BC patients with tumor grades 3 and 2, DCIS, and non-cancer individuals (P = 0.0005, AUC = 0.7419, threshold: 1.871 ng/ml). **C**, association between WFIKKN2 abundance and clinical stages, including stage 2/3, stage 1, DCIS, and non-cancer groups (P = 0.0009, AUC = 0.7190, threshold: 1.743 ng/ml). **D**, comparison of plasma WFIKKN2 levels between premenopausal and postmenopausal HER2+ BC patients (P < 0.0001, AUC = 0.7536, threshold: 1.871). **E**, correlation of WFIKKN2 plasma levels with TNM staging groups IIA/IIB, IA/IB, stage 0 (DCIS), and non-cancer controls. (P = 0.0016, AUC = 0.7103, threshold: 1.743 ng/ml). F, CA15-3 level in IDC patients and non-cancer individuals (P = 0.6, AUC = 0.5309, threshold: 9.259 U/ml). **G**, combined scores derived from multivariate logistic regression, with significantly higher values in IDC compared with non-tumor samples (P < 0.0001). Combined WFIKKN2 and CA15-3 abundance values in IDC patients and non-cancer individuals (AUC = 0.7384, threshold = 072.46%, specificity = 71.79%). WFIKKN2 was independently associated with IDC (OR = 2.97, 95% CI: 1.56–6.59, p = 0.0029), while CA15-3 did not reach significance (OR = 1.04, 95% CI: 0.97–1.13, p = 0.31) in the multivariate regression model. WFIKKN2 abundance in plasma of non-cancer individuals: M = 1.926 ng/ml. The optimal cut-off value was determined using the youden index (J = sensitivity + specificity −1) to maximize both sensitivity and specificity in the ROC analysis. The various AUC statistical calculations and sensitivity/specificity tables are provided in supplementary table S3 and supplementary table S4. The ELISA raw data and corresponding clinical information are presented in supplementary table S2. NC; non-tumor, IDC; invasive ductal carcinoma, DCIS; ductal carcinoma in-situ, TG; tumor grade, TS; tumor stage, POM, post-menopausal, PRM; pre-menopausal, TMN; tumor, node, metastasis. Statistical significance was determined by Kruskal–wallis where * = P < 0.05; ** = P < 0.01; and *** = P < 0.001 vs. non-cancer

Plasma WFIKKN2 levels were significantly higher in HER2+ BC patients with invasive ductal carcinoma (IDC) (M = 2.54 ng/ml) compared to controls (M = 1.93 ng/ml), with 77.8% sensitivity and 59.6% specificity (Fig. 2A). No difference was observed between patients with ductal carcinoma in situ (DCIS) and controls, indicating that WFIKKN2 is unsuitable for early detection. No difference in plasma WFIKKN2 level was observed in IDC patients with different grades and stages although the IDCs exhibited statistically higher WFIKKN2 levels compared to controls when samples were categorized by grade (Sensitivity 88.46%, Specificity 59.62) and stage (Sensitivity 87.10%, Specificity 51.92%) (Figs. 2B, 2C). Postmenopausal patients with IDC had significantly higher WFIKKN2 level (M = 2.674 ng/ml) relative to non-cancer controls (M = 1.926 ng/ml) with Sensitivity of 86.49% and Specificity of 52.62% (Fig. 2D). Finally, WFIKNN2 was found to be elevated significantly in TNM stages II A/B (M = 2.537 ng/ml) and I A/B (M = 2.533 ng/ml) compared to TNM 0 and non-cancer samples (Sensitivity = 86.67%, Specificity = 51.92%). No significant correlation was observed for patients with different nodal and ER/PR status (Fig. 2E). Our data suggest that aberrant WFIKKN2 expression is associated with early stages of cancer but not with pre-neoplasia.

CA15-3 showed 49% sensitivity and 80% specificity at 10 U/mL cutoff, and 5% sensitivity and 100% specificity at the clinical threshold (~30 U/mL) (Fig. 2F). The sensitivity of WFIKKN2 was higher than CA15-3 in both cases suggesting that WFIKKN2 is more suitable for cancer surveillance and recurrence monitoring [8, 9]. Combining WFIKKN2 and CA15-3 produced sensitivity of 72.46% and specificity of 71.79% (Fig. 2G). While this has a higher specificity than WFIKKN2 alone, the sensitivity of WFIKKN2 was reduced concomitantly.

In this study, WFIKKN2 was identified as a novel HER2 co-amplified gene with potential application as a blood-based biomarker for HER2+ breast cancer surveillance. While other HER2-coamplified genes such as WBP2 and STARD3 have been demonstrated to play roles in cancer biology and/or clinical applications [10, 11], these intracellular proteins are not amenable to non-invasive detection. In contrast, WFIKKN2 blood test is highly attractive due to its ease of implementation.

The role of WFIKKN2 in cancer remains to be investigated. WFIKKN2 binds and antagonizes growth differentiation factors GDF8 and GDF11, and interacts with BMP2 and BMP4 [12]. This limited information suggests a possible role of WFIKKN2 in reshaping growth factor signaling. Further studies are required to determine whether WFIKKN2 is oncogenic or merely a bystander.

While not ideal for diagnosis, the strong sensitivity of WFIKKN2 makes it superior to CA15-3 as a promising biomarker for surveillance and recurrence monitoring. Combining with CA15-3 improved WFIKNN2 specificity (72%) but reduced sensitivity (73%). Nonetheless, its use alongside other blood-based biomarkers could enhance broader clinical applications.

In conclusion, a blood test for WFIKKN2 could provide a cheaper, less invasive alternative to imaging for cancer monitoring.

## Electronic supplementary material

Below is the link to the electronic supplementary material.

**Supplementary Material 1:** Supplementary information S1

**Supplementary Material 2:** Supplementary Table S1

**Supplementary Material 3:** Supplementary Table S2

**Supplementary Material 4:** Supplementary Table S3

**Supplementary Material 5:** Supplementary Table S4

**Supplementary Material 6:** Supplementary Table S5

## Data Availability

The datasets used and analyzed during the current study are available from the corresponding author on reasonable request.

## Abbreviations

WFIKKN2: WAP, Follistatin/Kazal, Immunoglobulin, Kunitz and Netrin domain-containing protein 2
HER+ BC: HER2- positive breast cancer
BC: Breast cancer
Ch17q: Chromosome 17 q arm
CNV: Copy number variation
GDF: Growth Differentiation Factor
BMP: Bone Morphogenetic Protein
M: Mean
ELISA: Enzyme-linked immunosorbent assay
ER/PR+ BC: Estrogen receptor/progesterone receptor positive breast cancer
TNBC: Triple negative breast cancer
DCIS: Ductal carcinoma in-situ
IDC: Invasive ductal carcinoma
TNM: Tumor, node, metastasis
HPA: Human protein atlas
TCGA: The cancer genome atlas
AUC: Area under curve
TGF-β: Transforming growth factor-beta
STARD3: StAR-related lipid transfer domain protein 3
WBP2: WW Domain Binding Protein 2
