# Supplementary material for: WFIKKN2 is secreted and elevated in blood plasma of HER2-positive breast cancer patients – implications in cancer surveillance and recurrence monitoring
Source: Biomark Res. 2025 Nov 5;13:142. doi: 10.1186/s40364-025-00853-4 (PMC12590779; doi:10.1186/s40364-025-00853-4)

**Methods and material**

**Antibodies**

Human polyclonal anti-WFIKKN2 antibody (AF2070-GASP-1/WFIKKN2) was purchased from RnD systems-USA. Human monoclonal anti-HER2 antibody (2165-HER2/ErbB2 29D8) was from CELL SIGNALING TECHNOLOGY/USA. Mouse GAPDH mouse monoclonal antibody (AC002) was purchased from ABCLONAL TECHNOLOGY, USA. Rabbit Anti-Goat IgG H&L-HRP (ab6741) secondary antibody was from ABCAM/UK. Goat Anti-Mouse IgG (H&L) HRP(L35007) secondary antibody was purchased from SIGNALWAY ANTIBODY, USA.

**Cell lines and culture conditions**

SKBR3, MCF7, BT20, BT474, BT549, MDA-MB-231, MDA-MB-453, MDA-MB-468, T47D, and ZR-75-30 were obtained from Cell Bank of Chinese Academy of Sciences (No. 320 Yueyang Road, Xuhui District, Shanghai 200031, China). MCF7 was cultured in Dulbecco’s modified Eagle medium (C11995500BT-DMEM, Gibco, USA) and all other cell lines cultured in Roswell Park Memorial Institute-1640, RPMI-1640 (C11875500BT-Gibco, USA) medium. Both media were supplemented with 10% fetal bovine serum (FSP500-EXCELL, CHINA) + 1% Penicillin-Streptomycin (15140122-Gibco, USA) and incubated at 37°C in a humidified atmosphere with 5% CO_2_. All cell lines were authenticated using short tandem repeat DNA profiling by Jingke Biological Technology (Tsingke, Beijing No. 156, CHINA) and tested for mycoplasma contamination (LT07710-LONZA, Switzerland).

**Preparation of conditioned media**

To prepare serum-free conditioned media, cells were cultured at 37°C under a humidified atmosphere containing 5% CO₂ until they reached about 80% confluence. Following this, the cells were washed twice with D-phosphate-buffered saline (14190144-Gibco, USA) and incubated for 24 hours in serum-free RPMI/DMEM medium. After the 24-hour starvation period, the cells were checked visually to ensure they remained healthy. The media, now containing secreted proteins, was collected and following 10 minutes of centrifugation at 2000g was passed through a 0.22 μm filter (SLGPR33RB-Millipore, MA) to eliminate debris from dead cells and other impurities. The filtered media was then concentrated using 10000MWCO Amicon® Ultra Centrifugal devices (UFC901096-Millipore, MA) by centrifuging for 20 minutes at 4000g. The concentrated liquid was collected, and total protein concentration was measured using the BCA (23225-Thermo fisher, USA), with bovine serum albumin (BSA) serving as the standard.

**Reverse transcription and real-time PCR**

Cell lines were seeded in 6-well plates and total RNA extracted at 85% of confluency, using the MiniBEST Universal RNA Extraction Kit (9767-TAKARA, JAPAN). 500ng of RNA was reverse-transcribed with the HiScript II 1st Strand cDNA Synthesis Kit (R211-01-VAZYME, CHINA) employing both oligo(dT) and random hexamer primers. Quantitative real‐time PCR was carried out using Hieff UNICON® ColorGPS qPCR SYBR Green Master Mix (11188ES08-YEASEN, CHINA) according to the manufacturer’s protocol on qTOWER_3_G system (ANALYTIK JENA, Germany). The following primers were used: for WFIKKN2, forward 5′-TGGACTCCGGCACAATCCTGA-3′ and reverse 5′-CGTTGGGGCAGCACTTCTCAT-3′; for HER2, forward 5′-TGGCCTGTGCCCACTATAAG-3′ and reverse 5′-AGGAGAGGTCAGGTTTCACAC-3′. Relative gene expression was normalized to housekeeping gene GAPDH, forward 5′-TGCACCACCAACTGCTTAGC-3′ and Reverse 5′-GGCATGGACTGTGGTCATGAG-3′ using ΔΔC t method.

**siRNA sequences and transfection**

SiRNA sequences were purchased from Jingke Biological Technology (Tsingke, CHINA) The universal negative control siRNA sequences (NC) are: sense 5′-AUACGCGUAUUAUACGCGAUUAACGAC-3′, anti-sense 5′-GUCGUUAAUCGCGUAUAAUACGCGUAU-3′. The WFIKKN2 sequences are: sense 5′-CAACCGACGUGUCACAGAA-3′, anti-sense 5′-UUCUGUGACACGUCGGUUG-3′. SiRNAs were transfected using jetPRIME® transfection kit (101000046-Polyplus, FRANCE). To prove the specificity of WFIKKN2 antibody, 100 nmol of control and WFIKKN2-specific siRNA was transfected into 500,000 cells 100 mm plates. After 72 hours, the conditioned media was collected, total protein quantified, and the samples used for Western blot analysis.

**Western blot analysis**

Cells were washed twice with ice-cold PBS and lysed in ice-cold RIPA lysis buffer (Strong) (P0013B-BEYOTIME, CHINA) that contained (50mM Tris (pH 7.4), 150mM NaCl, 1% Triton X-100, 1% sodium deoxycholate, 0.1% SDS, and sodium orthovanadate, sodium fluoride, EDTA, leupeptin. Protease and phosphatase inhibitor cocktail (A32959- Thermo fisher, USA) were added separately and the cells incubated with lysis buffer on ice for 15 minutes to ensure complete lysis. The lysates were centrifuged at 20,000 × g for 20 minutes at 4°C, and the resulting supernatants were collected for Western blotting. Protein concentration in the samples was measured using the BCA (23225-Thermo fisher, USA), with BSA as the standard.

For Western blotting, equal amounts of protein were denatured in SDS sample buffer containing 100 mM β-mercaptoethanol, separated by SDS-PAGE, and transferred onto 0.45 µm PVDF membranes (IPVH00010, Sigma-Aldrich, USA) membranes. Coomassie-blue staining was performed as an indicator for loading control (P0017F- Beyotime, CHINA). The blots were incubated with BSA 5% (V900933-Sigma Aldrich, USA) for 1 hour at RT and probed with primary antibodies at 4°C overnight. Next day, blots were washed three times for 5 minutes each and incubated with horseradish peroxidase (HRP)-conjugated secondary antibodies for 1 hour with the membrane, Following After three additional washes (10 minutes each), the signals were developed using Ultra High Sensitivity ECL Kit Western Blotting Substrate (HY-K1005-MCE, USA) and visualized in GeneGnome XRQ system (Syngene, UK). Image processing and analysis were done using ImageJ software(1).

**Human clinical specimens**

Plasma samples were collected from 72 patients diagnosed with HER2-positive breast cancer at Peking Hospital, China, together with their matched clinical and pathological data. Samples were stored at −80°C until further use. Briefly, peripheral venous blood samples (8–10 mL per patient) were collected in EDTA-coated vacutainer tubes (367841-BD Biosciences, CHINA) before surgery and processed within 24 hours. Whole blood was diluted 1:1 with phosphate-buffered saline PBS (pH 7.4-Thermo Fisher Scientific, USA) and carefully layered over Ficoll-Paque PLUS density gradient medium (GE Healthcare) in a 15 mL conical tube. Centrifugation was performed at 400 × g for 30 minutes at room temperature with the brake disabled to ensure undisturbed layer separation. Following plasma collection, the distinct PBMC layer, located at the plasma-Ficoll interface, was aspirated and transferred to a new tube. To remove residual platelets and plasma components, PBMCs were washed twice with PBS via centrifugation (300 × g, 10 minutes). In addition, plasma samples from 52 clinically confirmed non-tumor control subjects were obtained. Whole blood samples were maintained at room temperature for 24 hours, after transferring to EDTA tubes, followed by centrifugation at 2000 × g for 10 minutes at 4°C, the resulting plasma was carefully isolated, aliquoted and stored at −80°C for subsequent analyses.

**Enzyme-Linked Immunosorbent Assay (ELISA)-WFIKKN2**

WFIKKN2 levels were quantified in 124 serum samples in triplicates, comprising 72 samples from HER2-positive DCIS and IDC patients and 52 from non-tumor controls, using the Human GASP-1/WFIKKN2 DuoSet ELISA kit (DY2070- R&D Systems, USA) according to the manufacturer’s protocol. Costar® 96-well high binding ELISA plate (9018- Corning, USA) were coated with capture WFIKKN2 antibody (provided in the kit, 2 μg/ml) diluted with PBS and incubated overnight at RT. The next day, wells were blocked with PBS containing PBS + 1% BSA (Dilution buffer) for 1 hour, followed by the addition of 100 µL of plasma samples + dilution buffer (1:1) to each well and incubated for 2 hours at RT. 100 μl of biotinylated detection antibody diluted with dilution buffer to a final concentration of 100ng/ml and incubated for another 2 hours. Subsequently, streptavidin-conjugated HRP diluted with dilution buffer to 1:200, substrate ((H2O2 (191201-SOUTHCHINA PHARMA, CHINA) + TMB G2412598-ALADDIN, CHINA)- 1:1), and stop solution (ABS9472-ABSIN, CHINA) were added sequentially, with each step incubated for 20 minutes at RT. Absorbance was measured at 450 nm using a TECAN microplate reader (Switzerland). Washing was done 3 times via quick aspiration with wash buffer (PBS + 0.05% Tween20 (9005645-Diamond, CHINA) after capture antibody, blocking, detection antibody, and streptavidin-conjugated HRP incubation.

**Enzyme-Linked Immunosorbent Assay (ELISA)-CA15-3**

CA15-3 levels were quantified in 98 serum samples in triplicates, comprising 54 samples from HER2-positive IDC patients and 39 from non-cancer individuals, using the Human CA15-3 ELISA Kit 96T (JM-7415H1, JIANGMEI, China) according to the manufacturer’s protocol.

Briefly, 20ul Serum samples were diluted with 80ul of sample diluent (Provided by the kit) and added to 96-well high-binding ELISA microplate, pre-coated with CA15-3 capture antibody (Provided by the kit). The plate was incubated for 30 minutes at 37 °C, then washed five times with 300 µL of wash buffer (Provided by the kit). Each wash followed by a 30-second incubation.

Next, 50 µL of HRP-labeled CA15-3 detection antibody (provided by the kit) was added to each well, followed by incubation for 30 minutes at 37 °C. After repeating the washing procedure, 100 µL of TMB substrate solution (1:1 mixture of solutions A and B, provided by the kit) was added to each well. Plates were gently shaken and incubated in the dark for 10 minutes at 37 °C. Finally, 50 µL of stop solution was added, and absorbance was measured at 450 nm using a TECAN microplate reader (Switzerland).

**Statistical analysis**

All experiments were conducted with triplicates, and results are presented as the mean ± standard deviation (SD). Comparisons between two groups were performed using the Mann–Whitney U test, while comparisons among multiple groups were assessed using the Kruskal–Wallis test. A P-value of <0.05 was considered statistically significant and indicated as follows: *P < 0.05; **P < 0.01; ***P < 0.001. All statistical analyses were performed using SPSS and GraphPad Prism software.

**Internal control and processing of ELISA data**

A normalization method was applied to standardize ELISA values across all assay plates. A plasma spike control was used for normalization across ELISA plates. To normalize the raw sample values across different ELISA plates, each sample was adjusted by multiplying the raw value by a normalization factor. This factor was calculated by dividing the average spike value across all plates by the spike value of each individual plate. Thus, the normalized sample value accounts for any variability between plates by standardizing measurements relative to the average spike control across the experiment.

**The** **somatic copy number variation analysis**

Copy number data generated by Affymetrix SNP 6.0 platform was taken from the TCGA database. The ‘nocnv.seg’ file which contains chromosome location and segment mean value for each tumor sample were collected to capture the somatic CNV. The GISTIC2 (2) with default parameters was used to obtain the focal copy number estimates. Those regions identified as “Amp” and q-value < 0.05 was selected as those significantly amplified. Next, the genome annotation information for protein coding genes from GENCODE (hg38) was obtained. The genome coordinate for individual gene was mapped to the focal CNV regions obtained by GISTIC2(3) in the genome to screen those with significant amplifications. The cancer gene list and EGFR pathway genes were obtained from the MSigDB database(4) and were then removed from the co-amplified genes.

**Shortlisting of the target genes**

We implemented 2 data-mining filters to remove genes that are known to be associated with cancer and HER2 signaling. This resulted in 42 genes. Further filtering was carried out using resources such as GeneCards (5) and ExoCarta (6) and exhaustive literature research to select genes and found 13 genes as secreted or predicted as secreted proteins. Only 2 genes remained with evidence of being present in circulating blood, and WFIKKN2 was selected for further investigation due to existing mass spectrometry data in HPA database that supported its presence in blood (7) .

**Multivariate analysis-Combination of WFIKKN2 and CA15-3**

To assess whether combining the two biomarkers improved sensitivity and specificity performance, a binary logistic regression model was fitted using cancer/non-cancer status as the dependent variable and the ELISA values of WFIKKN2 and CA15-3 as independent variables. The model was estimated by maximum likelihood. The fitted regression equation was:

$$Logit\left( p \right)= \beta0+ \beta1 \times WFIKKN2+\beta2 \times CA15-3$$

where *p* is the predicted probability of tumor. Regression coefficients were estimated as
β0=−1.294, β1=0.688, β2=0.016.
For each sample, the combined score was calculated as:

$$p= \frac{1}{1+e-( \beta0+ \beta1 \times WFIKKN2+\beta2 \times CA15-3)}$$

This probability value (Combined Score) was used as the combined biomarker metric for subsequent ROC analysis. ROC curves and AUCs were generated for WFIKKN2 alone, CA15-3 alone, and the combined score. Sensitivity and specificity at optimal cutoffs were determined using Youden’s index. All ROC analyses were performed in GraphPad Prism (Version 10.4.0).

**References**

1. Schneider CA, Rasband WS, Eliceiri KW. NIH Image to ImageJ: 25 years of image analysis. Nature Methods. 2012;9(7):671-5.

2. Mermel CH, Schumacher SE, Hill B, Meyerson ML, Beroukhim R, Getz G. GISTIC2.0 facilitates sensitive and confident localization of the targets of focal somatic copy-number alteration in human cancers. Genome Biology. 2011;12(4):R41.

3. Mermel CH, Schumacher SE, Hill B, Meyerson ML, Beroukhim R, Getz G. GISTIC2. 0 facilitates sensitive and confident localization of the targets of focal somatic copy-number alteration in human cancers. Genome biology. 2011;12:1-14.

4. Hänzelmann S, Castelo R, Guinney J. GSVA: gene set variation analysis for microarray and RNA-seq data. BMC bioinformatics. 2013;14:1-15.

5. Stelzer G, Rosen N, Plaschkes I, Zimmerman S, Twik M, Fishilevich S, et al. The GeneCards Suite: From Gene Data Mining to Disease Genome Sequence Analyses. Curr Protoc Bioinformatics. 2016;54:1.30.1-1..3.

6. Mathivanan S, Fahner CJ, Reid GE, Simpson RJ. ExoCarta 2012: database of exosomal proteins, RNA and lipids. Nucleic Acids Research. 2011;40(D1):D1241-D4.

7. Uhlén M, Karlsson MJ, Hober A, Svensson AS, Scheffel J, Kotol D, et al. The human secretome. Sci Signal. 2019;12(609).

**Supplementary figure’s Legends:**

Table S1: Table s1: Patients’ clinical and histopathological characteristic. ER; Estrogen receptor. PR; Progesterone receptor. IDC; Invasive ductal carcinoma. DCIS; Ductal carcinoma in-situ. NC, non-cancer subjects. N; number of cases.

Table S2: Table S2: ELISA raw data and sample clinical and pathological information

Table S3: Table S3: Statistical information for ROC analysis.

Table S4: Table S4: Threshold, sensitivity and specificity of plasma WFIKKN2 levels (ng/mL) with various histopathological parameters in HER2-positive breast cancer patients

Table S5: 42 HER2 co-amplified genes were identified that have not been previously reported in cancer.

Figure S1: A normalization method was applied to standardize ELISA values across all assay plates. A consistent plasma spike control was used for normalization. Fresh whole blood from a healthy donor was collected, centrifuged at 2000g for 10 minutes, and plasma was extracted, aliquoted, and stored at −4°C as a spike control. All ELISA plates contained the same spike control sample. To normalize the raw sample values across different ELISA plates, each sample was adjusted by multiplying the raw value by a normalization factor. This factor was calculated by dividing the average spike value across all plates by the spike value of each individual plate. Thus, the normalized sample value accounts for any variability between plates by standardizing measurements relative to the average spike control across the experiment.


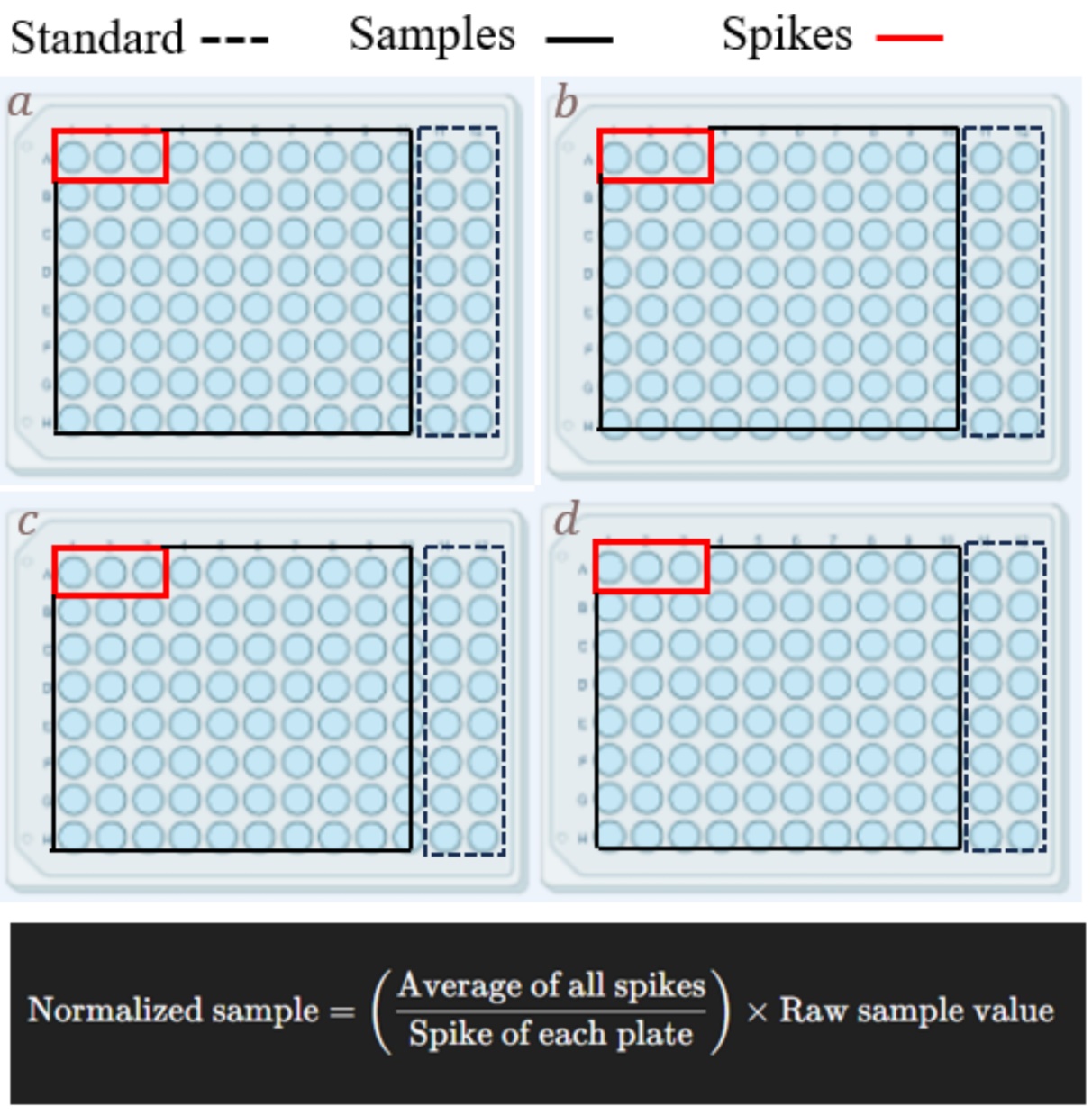


Figure S2: A, Serial dilution for WFIKKN2 protein in the conditioned media. 12 ug of protein observed as optimized amount of protein for evaluating WFIKKN2 across BC cell lines. Prove specificity for the WFIKKN2 antibody by siRNA mediated KD of WFIKKN2 antibody.


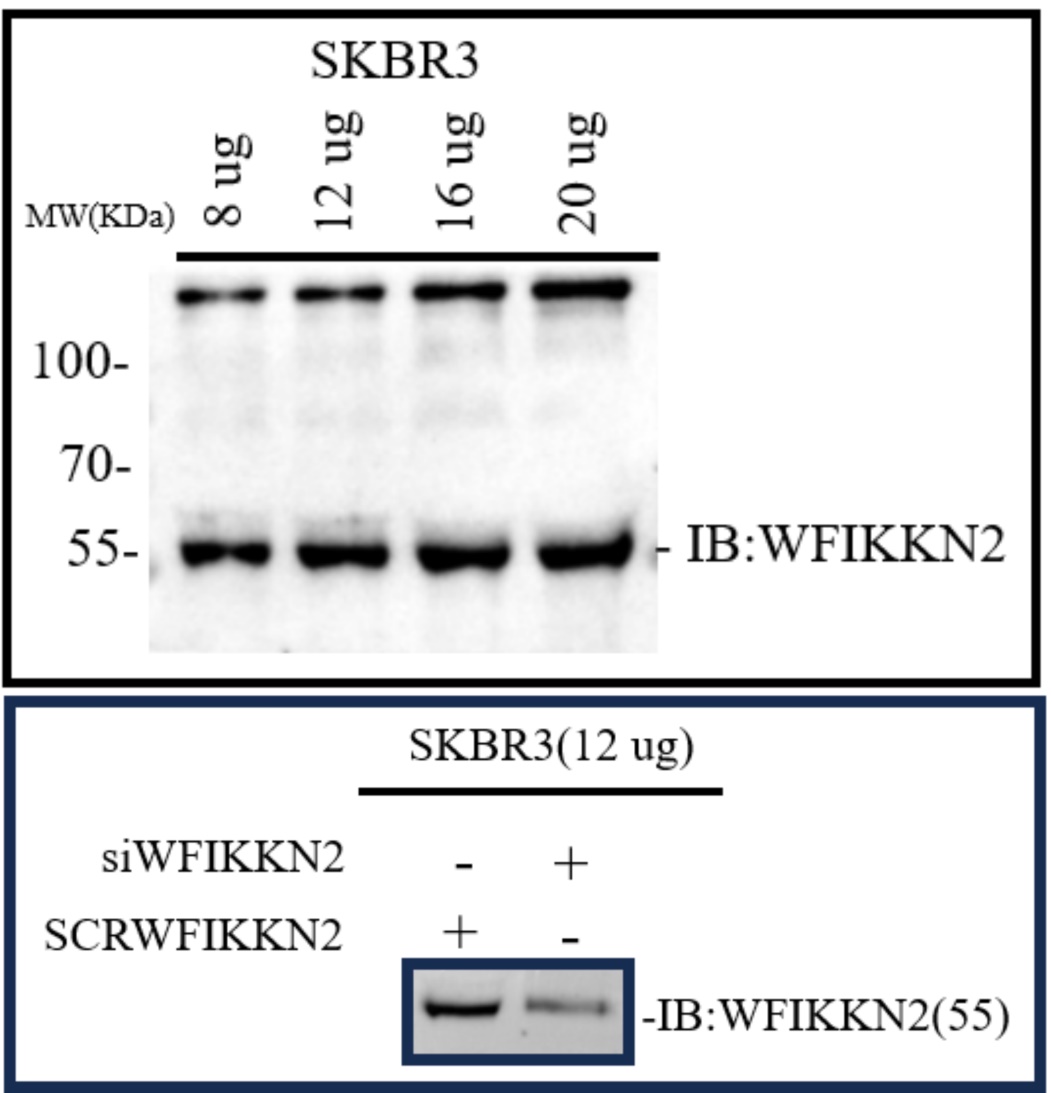

Supplement: Supplementary file 1 — Supplementary Material 1: Supplementary information S1 [file 40364_2025_853_MOESM1_ESM.docx]
