# Supplementary material for: WFIKKN2 is secreted and elevated in blood plasma of HER2-positive breast cancer patients – implications in cancer surveillance and recurrence monitoring
Source: Biomark Res. 2025 Nov 5;13:142. doi: 10.1186/s40364-025-00853-4 (PMC12590779; doi:10.1186/s40364-025-00853-4)
Supplement: Supplementary file 2 — Supplementary Material 2: Supplementary Table S1 [file 40364_2025_853_MOESM2_ESM.docx]

Table s1: Patients’ clinical and histopathological characteristic.

| **Characteristics** | **Description** | **N (%)** |
| --- | --- | --- |
| Age | Median | 58 |
|  | Range | 27 to 90 |
|  | <50 years old | 36(50) |
|  | ≥50 years old | 36(50) |
| Nature of samples | DCIS | 18(25) |
|  | IDC | 52(72.22) |
| Menopausal status | Pre-menopause | 25(34.72) |
|  | Post-menopause | 47(65.27) |
| ER status | Positive | 37(51.38) |
|  | Negative | 35(48.61) |
| PGR status | Positive | 23(31.94) |
|  | Negative | 49(68.05) |
| HER2 status | 3+ | 61(84.72) |
|  | 2+ (FISH) | 11(15.27) |
| Tumor grade | 1/2 | 29(40.27) |
|  | 3 | 43(59.72) |
| Tumor stage | T0(In-situ) | 18(25) |
|  | T1 | 23(31.94) |
|  | T2/3 | 31(43.05) |
| Node stage | N0 | 52(72.22) |
|  | N1/2 | 20(27.77) |
| Overall TNM stage | 0 | 18(25) |
|  | IA/B | 20(33.33) |
|  | IIA/B | 30(41.66) |
| Total | - | 72(100) |
| NC subjects | | |
| Age | Median | 52 |
|  | Range | 40-75 |
|  | <60 | 38(73) |
|  | >60 | 14(27) |
| Total | - | 52(100) |

Table legend: Table legend: ER; Estrogen receptor. PR; Progesterone receptor. IDC; Invasive ductal carcinoma. DCIS; Ductal carcinoma in-situ. NC, non-cancer subjects. N; number of cases.
