# Supplementary material for: WFIKKN2 is secreted and elevated in blood plasma of HER2-positive breast cancer patients – implications in cancer surveillance and recurrence monitoring
Source: Biomark Res. 2025 Nov 5;13:142. doi: 10.1186/s40364-025-00853-4 (PMC12590779; doi:10.1186/s40364-025-00853-4)
Supplement: Supplementary file 5 — Supplementary Material 5: Supplementary Table S4 [file 40364_2025_853_MOESM5_ESM.docx]

| **Type of comparison** | **Threshold value (ng/ml)** | **Sensitivity%** | **Specificity%** |
| --- | --- | --- | --- |
| IDC vs non-tumor | 1.871 | 77.78 | 59.62 |
| TG3 vs non-tumor | 1.871 | 88.46 | 59.62 |
| TS2 vs non-tumor | 1.743 | 87.1 | 51.92 |
| POM vs non-tumor | 1.871 | 86.49 | 52.62 |
| TMN IIA/B vs non-tumor | 1.743 | 86.67 | 51.92 |

Table S4: Table S4: Area under curve analysis. Threshold, sensitivity and specificity of plasma WFIKKN2 levels (ng/mL) with various histopathological parameters in HER2-positive breast cancer patients.

Table legend: NC; Non-tumor control, TG; Tumor grade, TS; Tumor stage, POM; Post-menopausal status.
